# Tooth Autotransplantation as an Alternative Biological Treatment: A Literature Review

**DOI:** 10.7759/cureus.30491

**Published:** 2022-10-19

**Authors:** Kadambari Kakde, Rajanikanth K

**Affiliations:** 1 Oral and Maxillofacial Surgery, Sharad Pawar Dental College, Datta Meghe Institute of Medical Sciences, Wardha, IND

**Keywords:** conservative treatment, dental trauma, third molar, extraction, autotransplantation

## Abstract

Tooth autotransplantation is the treatment modality in which a tooth is transferred from one site to another in the same person. This technique has a history of centuries. However, it is not well-known or well-documented. Essential aspects of the clinical procedure, criteria for performing tooth autotransplantation, advantages, disadvantages, and complications are also discussed in the review. It has been a public health concern considering the prevalence of dental trauma in children, the financial burden of treatment, and the long recovery times associated with dental injuries. There is evidence that tooth autotransplantation is an effective method of restoring missing teeth, particularly for growing children. Even if autotransplantation fails, the soft tissue and bone conditions would likely still be suitable for subsequent implant treatment. Appropriate patient and tooth selection are essential to the technique's success. Other prognostic factors are also discussed. The findings from the available literature suggest that tooth autotransplantation is a viable and cost-effective technique. This paper discusses the literature and protocols the authors implemented for autotransplantation of the tooth.

## Introduction and background

The transfer of a tooth within the same individual is known as tooth autotransplantation [1]. The procedure of tooth autotransplantation originated through allotransplantation when two individuals swapped their teeth. The work of surgeon Ambroise Pare is documented as far back as 1594 when royal families received teeth transplants. John Hunter performed the first successful tooth transplant in 1772 [2]. The transmission of disease and immune compatibility were not cognized or considered. Autografts, now called autotransplantation, played an important role in later work. Several centuries have passed since autotransplantation was developed, but the first clinical cases were reported in the 1950s. This field was first investigated by surgically enucleating unerupted or partially erupted third molars [3,4]. This method involves moving a tooth from one location to another where its functional and esthetic properties are better suited. It is not unusual to use third molars to replace posterior teeth when they are severely damaged and cannot be rehabilitated [5]. A premolar or canine is typically used as a donor tooth in cases of oral infection in the anterior maxilla [6-8]. More than two-thirds of children suffer tooth trauma before the age of 16 [9-12]. The loss of permanent teeth occurs in 7-8% of these dental traumas. [13,14]. A typical application of the technique is in pediatric dentistry, replacing poorly prognosed or absent front teeth with partially developed premolars [15]. Third molars have been autotransplanted for over half a century and provide a good alternative for replacing first-or second-molars with a poor prognosis [16], regardless of the cause, such as caries, periodontal disease, or another circumstance. Tooth autotransplants are typically recommended for people with tooth loss due to trauma or dental caries, missing teeth, impacted teeth, tumors, treatment-related injuries, and teeth with a poor prognosis [17], the last group being the most prevalent in clinical studies related to third molars. Moreover, a wide range of indications have been discussed recently as an alternative to dental implants, as a space maintainer, in hypodontia, in regional odontodysplasia, in oroantral communication, where bone generation is needed, in clefts, and also in jaw reconstruction [18]. This paper aimed to review treatment alternatives when autotransplantation is used to replace teeth [19] damaged by trauma, mainly restoring the aesthetic appearance and functional occlusion and inclination of the incisors.

## Review

Essential aspects in the clinical procedure

A tooth as a donor is generally selected from the same quadrant as the neighboring teeth since these tend to be morphologically similar [20]. In most cases, upper third molars can also be suitable donors to replace lower molars. It is essential to extract donor teeth in as minimally invasive a manner as possible to prevent excessive destruction of the periodontal ligament (PDL). In the meantime, the much-needed tooth can be placed on gauze soaked in saline solution or left in the donor site until the site is ready for the transplant [21-22]. But it is always better to prepare the transplant bed before the potential donor tooth is extracted. The ideal solutions for placing the extracted tooth are saliva and its variants, milk and derivatives, Hank's Balanced Salt Solution (HBSS) and its variants, and saline and its variants. However, the literature suggests milk is the most viable option regarding PDL cell viability, ease of availability, and cost-effectiveness, followed by HBSS [23]. Some modifications should be made to the socket to accommodate the donor's tooth. The third molar with convergent roots may require the removal of the intraalveolar septum to provide proper seating. Rongeurs might be used to remove this manually, or low-speed round burs with copious irrigation may assist in removing it surgically [24]. A large amount of saline irrigation prevents thermal damage [25-26]. The extracted tooth is transplanted. The implanted tooth must be stabilized after it is placed. To avoid occlusal trauma, the transplanted tooth should be placed in an infraocclusal position. Stabilization can be done using various methods like splinting, wires, composite splints, etc. A time-to-time evaluation for occlusion should be done to check the drifting of the transplanted tooth.

Criteria to perform autotransplantation

A person for whom autotransplantation treatment is being planned should be physically fit. Even a donor tooth should be healthy and its physical characteristics must match the recipient tooth without compromising occlusion. A tooth with an incompletely formed root with an incomplete apex (an apex greater than 1 mm across) would be much more favorable [27]. However, autotransplantation of mature teeth with complete root formation can be a predictable treatment and is not necessarily associated with poor prognosis [28]. A tooth must be removed without causing any damage to Hertwig’s epithelial root sheath (HERS), the PDL, and the developing tooth. All the procedures must be done adhering to the principle that the extra-alveolar time of the transplanted tooth should be a minimum. Some authors state that it is vital to minimize the extra-alveolar time of the donor's tooth as much as possible to preserve the periodontal ligament and maintain Hertwig’s epithelial root sheath to reduce complications [3,29]. The tooth to be transplanted should be placed in a fresh socket rather than one prepared for transplanting the tooth. The patient should maintain good oral hygiene following transplantation to avoid trauma.

Advantages and disadvantages

The advantages and disadvantages of autotransplantation are described in Table 1.

**Table 1:** The advantages and disadvantages of autotransplantation.

Advantages	Disadvantages
A better alternative to removable or fixed prostheses.	If the root resorbs and the root attachment diminishes, the tooth will fall out.
Preparation of adjacent teeth is not needed.	Poor oral hygiene can cause attachment loss, which leads to failure of treatment.
Cost-effectiveness	The restored tooth can not be used.
Proper thermal and proprioception responses.	Cannot be done in an infected site.
Can be moved orthodontically.	The infraocclusal position of the transplant could necessitate further procedures, e.g., orthodontic and/or prosthodontic treatment.
Normally functioning periodontium.	Endodontic treatment would be necessary if the pulpal tissues failed to heal.
Preserve the alveolar bone.	

Complications

Ankylosis and root resorption are the most probable complications.

Discussion 

By reviewing the literature, D Ong et al. [3] wish to illustrate how autotransplantation might benefit patients with severely compromised teeth and raise public perception of it as a possible treatment for severely damaged teeth. A patient with orthodontic space closure that isn't predictable or practical can undergo autotransplantation, replacing a healthy immature donor tooth instead of a prosthetic restoration. Providing the proper parameters are met and highly experienced surgeons are consulted, it may be possible to achieve significant cost-effective benefits with autotransplantation of teeth in growing patients. Through the application of conical beam computed tomography (CBCT) imaging and enhanced speed of three-dimensional (3D) image modeling, this procedure is expected to have higher success rates and greater longevity, as the template produced is highly accurate and the donor's tooth is exposed for less time outside the mouth. There is nothing new about tooth autotransplantation. Through future technological advancement and improved clinician awareness, autotransplantation might become a more widely available option and have a much better chance of becoming a realistic treatment option for adolescents.

In the study by Vishwanath et al. [30], a lost central incisor was replaced successfully by autotransplantation in combination with orthodontic treatment in a growing child who had previously undergone facial trauma. A unique aspect of their case was that autotransplantation was performed on a person who did not fit the standard criteria. In traditional dentistry, dental autotransplantation is considered when there is a need to increase the space in the mouth due to crowding or proclined teeth. Despite the absence of strict guidelines for autotransplantation, there is no reason that orthodontic professionals cannot choose this procedure since we live in an era that includes tools such as 3-D imaging, CBCT, and temporary anchorage devices that can enhance predictability. In young patients with traumatic dental injury (TDI), the treatment can be unpredictable, expensive, and last for the rest of that person's life; therefore, they should minimize unwanted consequences as much as possible [31]. Various treatment options are available to patients, including removable prostheses and conventional fixed prostheses, dental implant-supported crowns, autotransplantation, and simple orthodontic space closure through replacement teeth. Space maintenance is usually required with this treatment modality in a growing child. In this period, there is constant remodeling of the alveolar bones on the buccal and palatal surfaces, possibly requiring alveolar bone grafts and leading to a higher treatment expense. In addition, a study over a long period [32] suggested that it will not be necessary to curtail vertical growth as well as predicted, with adult patients manifesting growth patterns to the same extent as adolescents. Teenagers with the above concerns may benefit from autotransplantation of teeth as a viable treatment option. We can recommend this treatment option for young patients who are missing maxillary incisors due to an effective transplant of a premolar surrounded by healthy tissue. An autotransplanted tooth is maintained with a fully functioning PDL and, therefore, can induce bone formation and allow continual movement while growing, restoring normal alveolar development. It is also possible that autotransplanted teeth show widely varying success and survival rates among different studies. When osseointegrated single-tooth implants were compared with autotransplanted teeth, the survival rates were comparable [33]. Over five years, Czochrowska et al. [34] followed up 100 autotransplanted teeth with a 90% survival rate and five single-tooth implants with a 94.5% survival rate.

Vasiliki and coworkers [35] explored the healing of pulp tissues and PDL in a pediatric population along with the association between these processes and various factors determining prognosis. The combination of clinical and radiological indicators was typically used to assess the success of autologous transplants. Studies have established and used several criteria when defining success and survival. Treatment success was determined by the extent to which external root resorption occurred and other factors influencing transplantation outcomes, such as pulp and PDL healing. Ultimately, the success of a tooth transplant is affected by various factors, with PDL healing being the most important. This is based on the fact that effective endodontic treatment can still provide good results in treating the affected tooth even if the pulp fails to heal. In contrast, if the post-transplant healing doesn't occur as planned, the tooth's long-term prognosis is compromised. When transplantation was performed, the subjects had a median age of 13.24±2.0 years. When a child is between 12 and 14 years of age, the first and second premolar roots develop completely. A premolar that has developed sufficiently long roots and a desired root-to-crown ratio at the time of autotransplant is an ideal candidate because it still has enough room at the apex for revascularization. The study excluded participants who had a shorter follow-up period than 12 months. Most of the healing would be completed in 12 months. This study identified the possibility of pulp healing in 39 out of 75 teeth with open apices. In 76.9% of the cases (n = 30), pulp revascularization was evident. The key indicators for diagnosing pulp survival were partial pulp canal obliteration and continued root growth. 73.5% of cases with evidence of revascularization at six months were found to have partial pulp canal obliteration at that time, and 100% of cases had full pulp canal obliteration 12 months after the transplant. Radiographic signs of obliteration of the pulp canal on follow-up after half a year of surgery have been reported. The overall success of tooth transplantation was 87.6%, and the survival rate was 94.4% [35].

In their review of 23 cases, Plakwicz et al. [36] show that pulp healing has occurred in most samples, which is 88.10%, leading to sustained root development with pulp canal obturation and continued root development with pulp canal obliteration in several cases. During transplantation, root formation was directly associated with the probability of pulp healing, which was identified as a prognostic factor. The authors determined that a diameter of more than one millimeter is associated with a lower risk of pulp necrosis based on their correlation between apical foramen size and pulp healing [36]. Teeth with wider apices and mature roots are more likely to revascularize more readily as compared to teeth with narrow apices. The vascular and nerve supply to the pulp is severed during autotransplantation. During the surgical operation, this causes substantial damage to the architecture and function of the pulp. Healing occurs after transplantation due to revascularization, which restores the pulp's blood and nerve supply. Thus, teeth with incomplete root development have a better chance of healing after transplantation since their wider openings improve vascularity. The results of this study by Plakwicz [36] agree with those of a recent systematic review, which concluded that despite poor quality studies, the real success of the procedure could be explained by the stage of root development [36]. A small percentage of cases with complete root development in this study demonstrated good healing. As for patients with root resorption, it was controlled with active endodontic treatment. Most of the cases in this study that had fully developed roots showed favorable healing. In addition, the surgical procedure is more challenging in places with poor bone quality. When the donor tooth is extracted and is exposed for an extended period, the exposed fibers and extra-alveolar time can cause damage to the cementum, making it more susceptible to osteoclastic activity. There was also a significant association between the condition of the alveolar bone at the donor site and favorable healing of PDLs. It seems essential to consider this factor from a clinical perspective, although previous studies have not reported on it as a prognostic factor [36]. A crucial clinical variable is the vertical and bucco-palatal height of the bone since both these dimensions are vital in allowing the transplanted tooth to be placed into a good bone, thereby promoting a successful healing outcome. It would also be better for aesthetics to have adequate vertical bone height. This study found that most recipients had acceptable bone levels because bone level maintenance was considered during interdisciplinary planning and decoration procedures were performed promptly when required. The specialists can sometimes delay the referral process, thus causing the bone to already be atrophying by the time the referral is received [36]. Because of the high levels of patient satisfaction and acceptance of this new technique, the authors anticipate that it may become standard therapy for traumatic tooth loss or agenesis in children and adolescents, serving as a "transitional technology" until other well-known follow-up treatments have been developed [37].

## Conclusions

There is not much literature on third-molar autotransplantation. Published studies, however, indicate a high rate of success. Patients should carefully consider the advantages and disadvantages of autotransplantation before making an informed decision about their treatment. As adjacent teeth do not need to be prepared, this is a more desirable option than traditional prosthetics. It is also more cost-effective, despite surgical methods' limitations. A tooth may also be lost because of adverse consequences, including clinical attachment loss and root resorption. For autotransplantation to be successful, selecting patients carefully and monitoring them for an extended period is essential. More than one study found a survival rate of more than 90%. The idea of autotransplanting teeth is not new. When physicians have an increased understanding of this treatment option, autotransplantation might become a sensible and practical treatment option for teenage patients who are deemed fit for it.
